# Supplementary material for: Proteomic changes in the human cerebrovasculature in Alzheimer's disease and related tauopathies linked to peripheral biomarkers in plasma and cerebrospinal fluid
Source: Alzheimers Dement. 2024 May 7;20(6):4043–65. doi: 10.1002/alz.13821 (PMC11180878; doi:10.1002/alz.13821)
Supplement: Supplementary file 1 — Supporting Information [file ALZ-20-4043-s001.pdf]

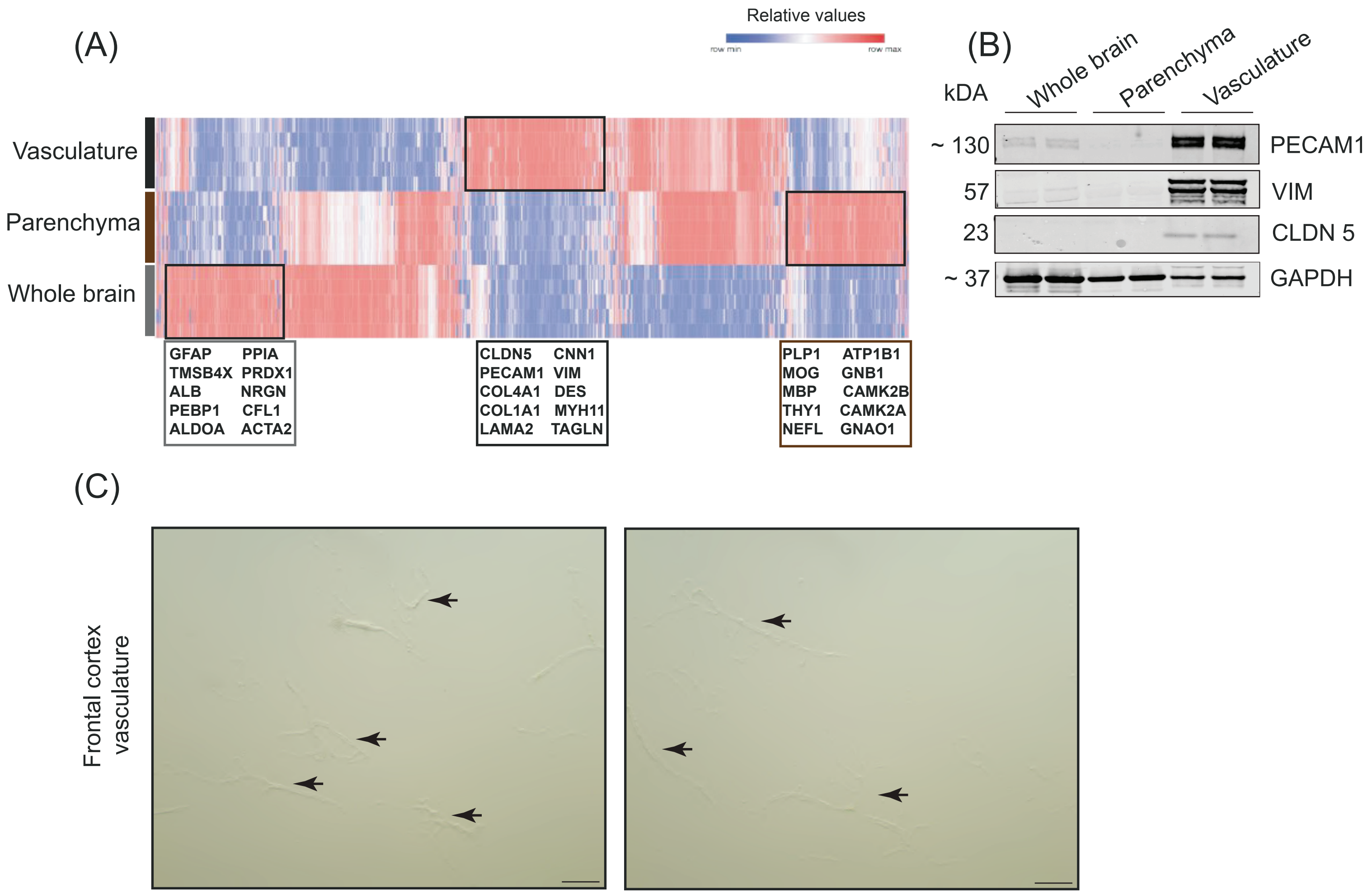

Supplemental Figure S1. Establishment of the method of cerebrovascular isolation.
(A) Label free mass spectrometry analysis of proteins enriched in whole brain, parenchyma, and isolated cerebrovasculature. Red indicates proteins increased and blue indicates proteins decreased in each brain fraction. (B) Western blot analysis shows enrichment of vascular-specific proteins in the vascular fractions compared to whole brain and parenchyma. (C) Microscopy images showing the purity of isolated vasculature. Scale bar, 100 μm.
