# Supplementary material for: Proteomic changes in the human cerebrovasculature in Alzheimer's disease and related tauopathies linked to peripheral biomarkers in plasma and cerebrospinal fluid
Source: Alzheimers Dement. 2024 May 7;20(6):4043–65. doi: 10.1002/alz.13821 (PMC11180878; doi:10.1002/alz.13821)
Supplement: Supplementary file 2 — Supporting Information [file ALZ-20-4043-s003.pdf]

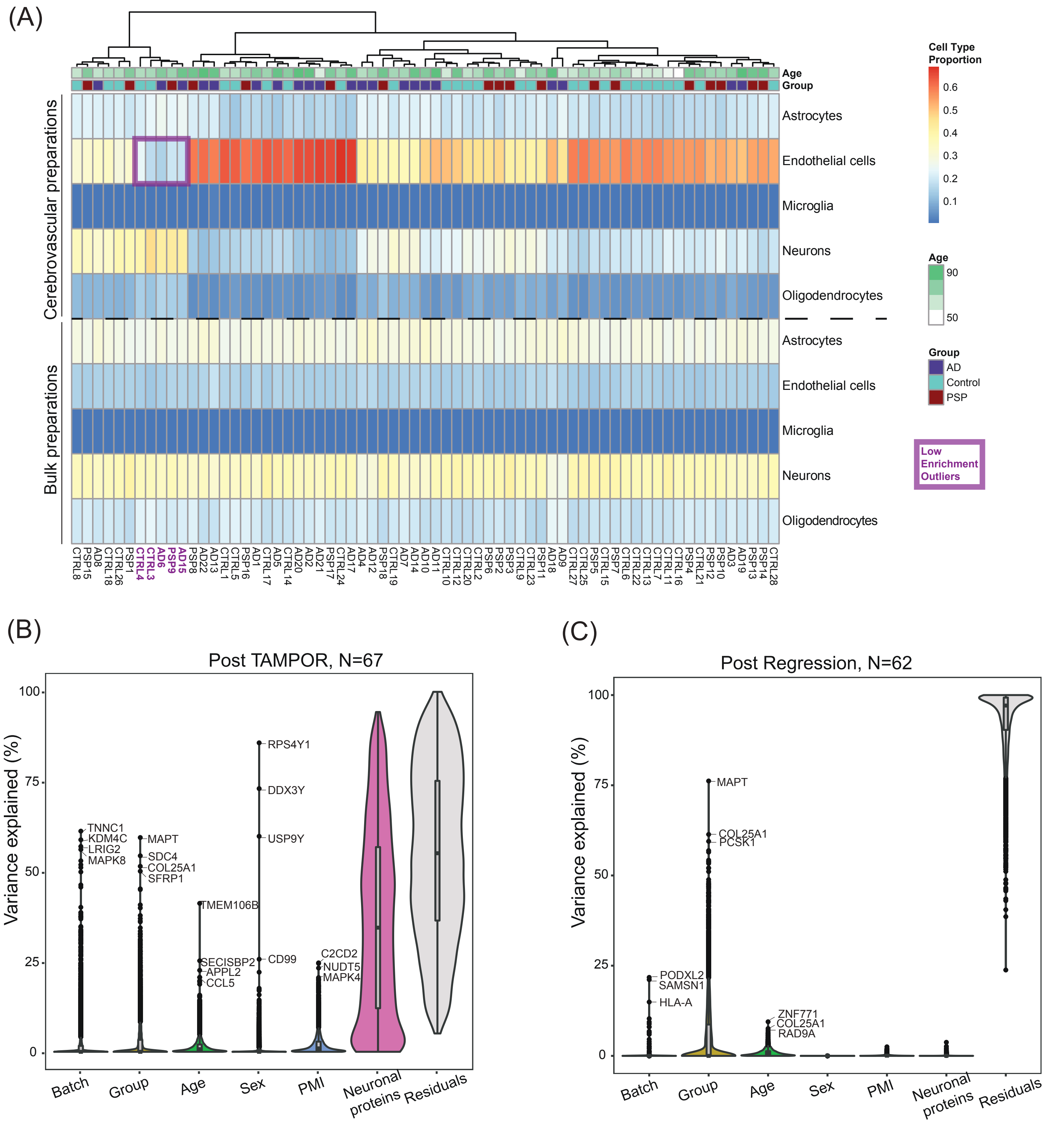

Supplemental Figure S2. Cell type proportions in the vascular and bulk proteomes. (A) Heatmap showing cell type proportions in the vascular proteome (above dashed line) and bulk proteome (bottom). Proportions of the five cell type total one, and the five cell types are defined via reprocessing of Darmanis[73], Nowakowski[31], and Zhong single-cell datasets as curated with the EnsDeconv R package, which calculated cell type proportions by ensemble deconvolution of each proteome[74]. Dark red and orange indicate high enrichment of endothelial proteins to proportions above 70 percent, and dark blue indicates the lowest enrichment of cell-specific proteins in the preparations. The purple box outlines a cluster of outliers in the vascular preparations with very low enrichment of endothelial markers. (B) Variance partition plots were used to visualize the percent variance of each protein in the data set co-varying with batch, group, age, sex, postmortem interval (PMI), and neuronal proportions across case samples. (C) Following outlier removal, the matrix was subjected to the bootstrap regression to remove the variance due to age, sex, PMI, and neuronal cell type proportions.
