# Supplementary material for: Proteomic changes in the human cerebrovasculature in Alzheimer's disease and related tauopathies linked to peripheral biomarkers in plasma and cerebrospinal fluid
Source: Alzheimers Dement. 2024 May 7;20(6):4043–65. doi: 10.1002/alz.13821 (PMC11180878; doi:10.1002/alz.13821)
Supplement: Supplementary file 3 — Supporting Information [file ALZ-20-4043-s007.pdf]

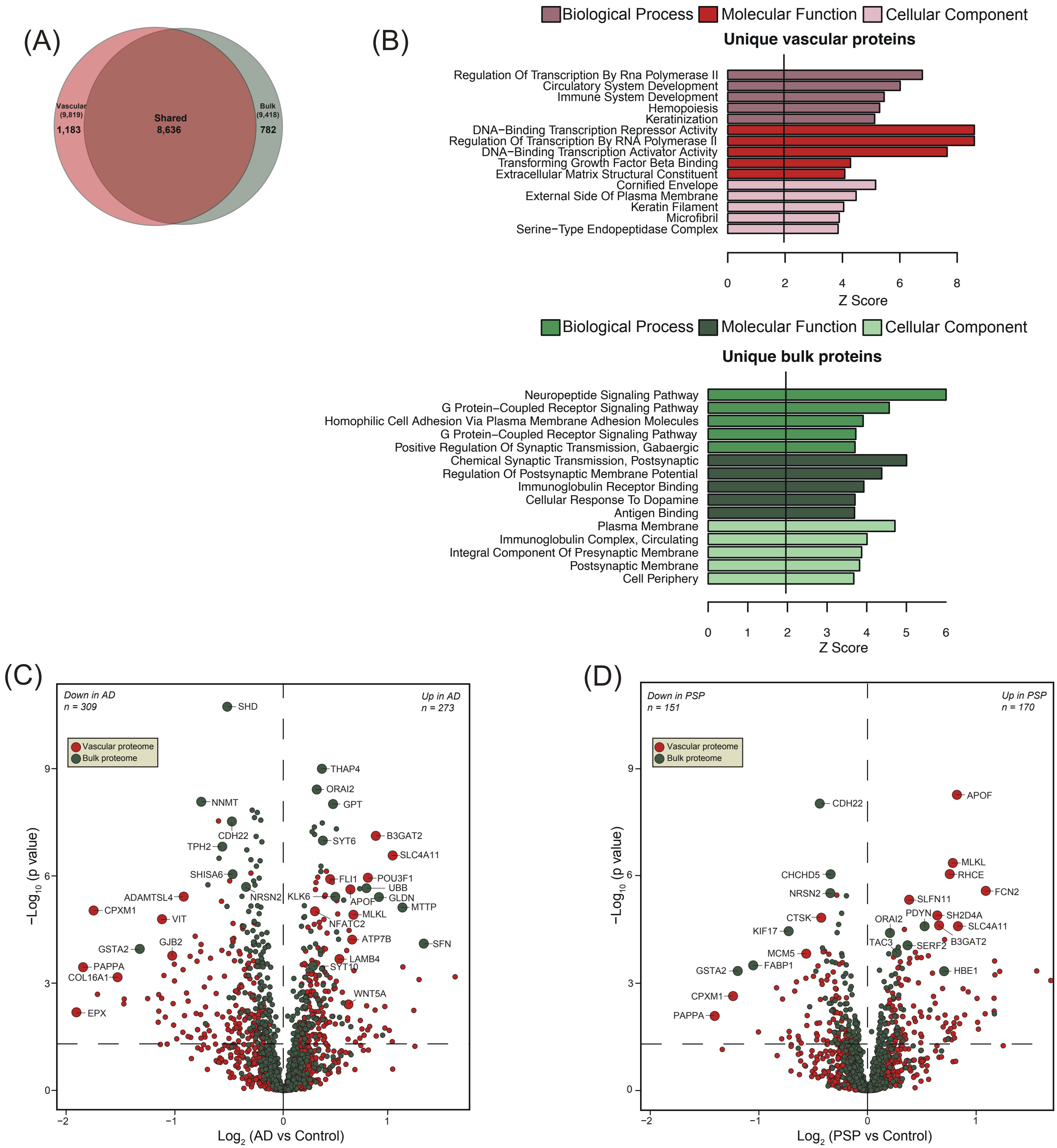


Supplemental Figure S3. Differentially abundant proteins unique to vascular and bulk proteomes reveals specific discriminators. (A) Venn diagram of shared and unique proteins discovered in vascular and bulk fractions; counts shown are for unique gene products, in distinction from total protein isoforms. (B) GO terms enrichment analysis was used to represent biological processes, molecular function, and cellular component for unique vascular or bulk proteins. (C-D) Proteins unique to either vascular fraction or bulk proteome were input for statistics underlying volcano plots displaying differential abundance of 582 proteins between Control and AD (C) or 321 proteins between Control and PSP (D). The x axis shows the log2 fold change, while the y axis represents -log10 statistical p value calculated for all proteins between pairwise vascular and bulk group comparisons in AD (C) and PSP (D). One-way ANOVA was performed followed by Tukey's post-hoc test for each pairwise comparison vs. control and imprecise Tukey p values below $10^{-8.5}$ were replaced with pairwise two-sided unequal variance T-tests' p values corrected for multiple tests by Bonferroni correction. Proteins are colored based on the proteome membership (red indicates vascular proteome and green indicates bulk proteome).
