# Supplementary material for: Proteomic changes in the human cerebrovasculature in Alzheimer's disease and related tauopathies linked to peripheral biomarkers in plasma and cerebrospinal fluid
Source: Alzheimers Dement. 2024 May 7;20(6):4043–65. doi: 10.1002/alz.13821 (PMC11180878; doi:10.1002/alz.13821)
Supplement: Supplementary file 4 — Supporting Information [file ALZ-20-4043-s002.pdf]

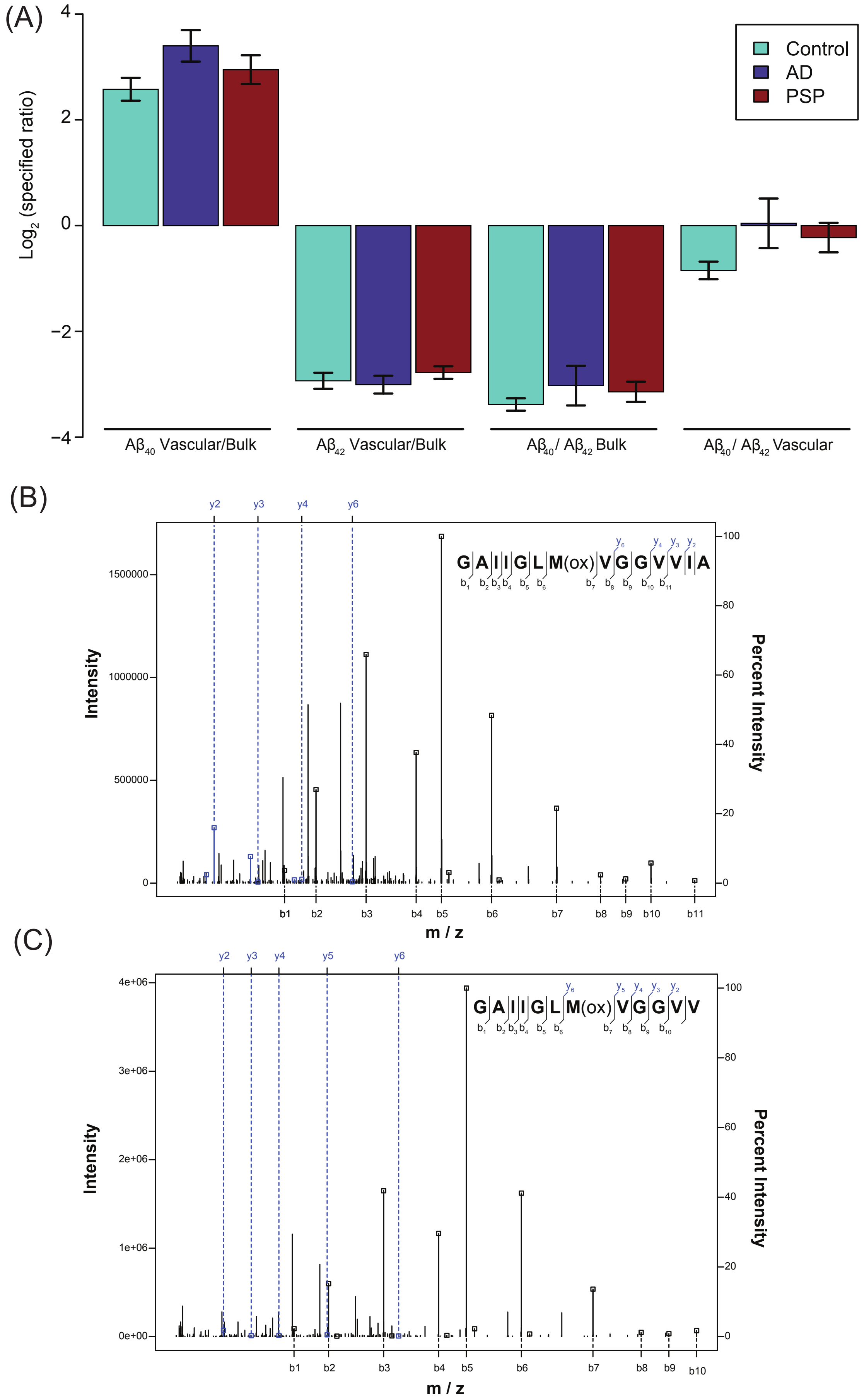

Supplemental Figure S4. Aβ40 is predominantly found in the brain vascular fraction. (A) MS-quantified levels of the C-terminal tryptic Aβ40 and Aβ42 species in the vascular and bulk fractions. (B) MS/MS spectrum of C-terminal tryptic peptide of Aβ42 with annotation of matched B and Y ions as indicated. (C) MS/MS spectrum of the cognate peptide for Aβ40.
