# Supplementary material for: Proteomic changes in the human cerebrovasculature in Alzheimer's disease and related tauopathies linked to peripheral biomarkers in plasma and cerebrospinal fluid
Source: Alzheimers Dement. 2024 May 7;20(6):4043–65. doi: 10.1002/alz.13821 (PMC11180878; doi:10.1002/alz.13821)
Supplement: Supplementary file 5 — Supporting Information [file ALZ-20-4043-s004.pdf]

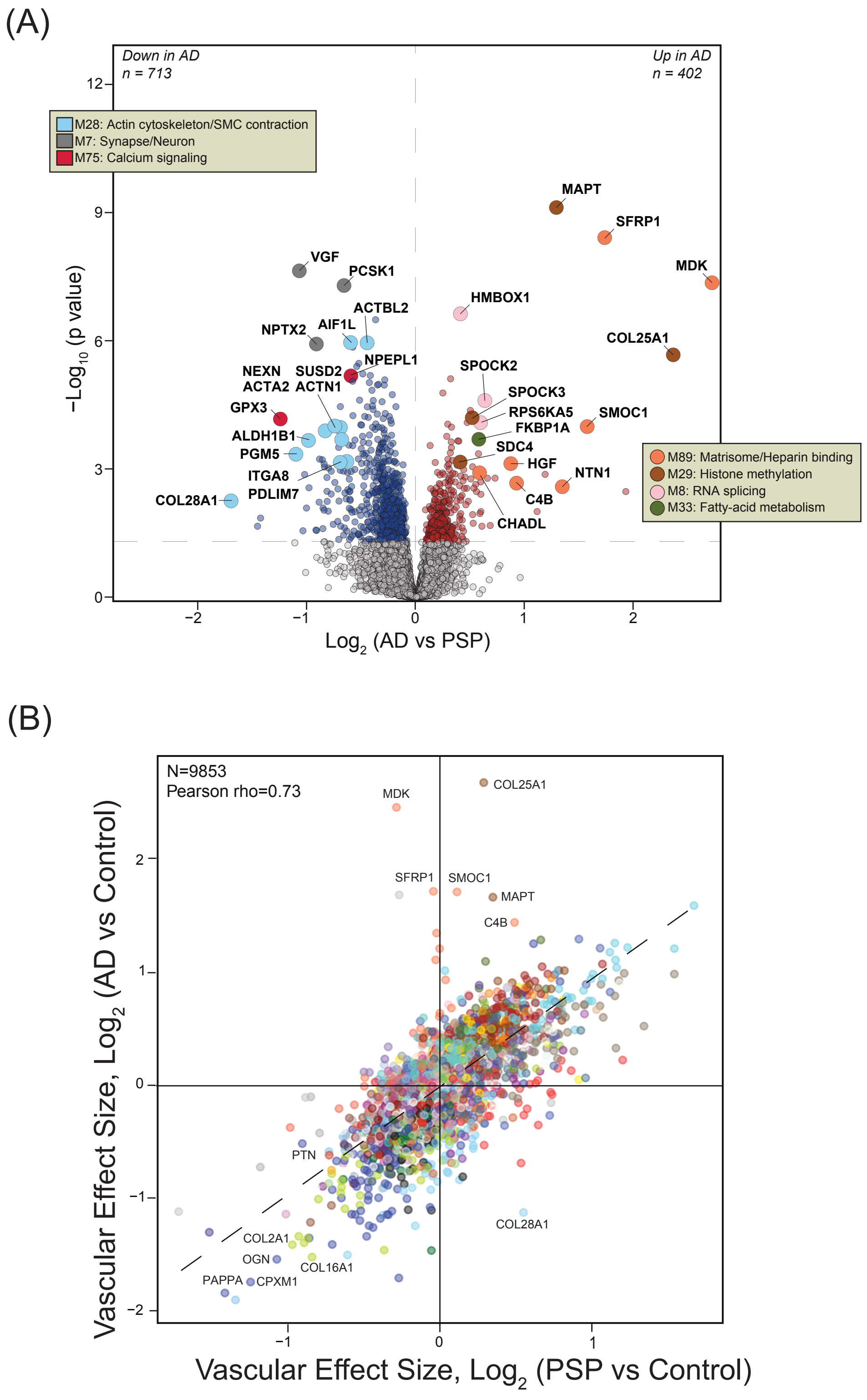

Supplemental Figure S5. Amyloid-associated changes in cerebrovasculature drive distinct proteomic signatures in AD and PSP. (A) Volcano plot showing the log2 fold change (x axis) and -log10 one-way ANOVA with Tukey p value (y axis) for differentially changed proteins between pairwise AD and PSP comparison. Tukey *p* values below $10^{-8.5}$ were recalculated as Bonferroni-corrected two-tailed unequal variance t-test *p* values. Proteins are shaded based on their module membership colors. (B) Scatter plot of the cerebrovascular proteome log2 effect size of the PSP group compared to unimpaired healthy Controls (x) versus the log2 effect size of the AD group compared to Controls (y). Highlighted proteins showcased disease-specific signatures altered in AD and PSP.
